# Histochemical and histometrical changes induced by the rust fungi *Kuehneola uredinis* in distinct blackberry (*Rubus* spp.) cultivars

**DOI:** 10.1007/s00709-026-02230-w

**Published:** 2026-06-17

**Authors:** Filipe R. Valeriano, Ivana P. de Sousa-Baracho, Rayssa R. Marquesine, Karoline S. de Sousa, Caíque M. de Abreu, Cássia M. Cabral, Cleber J. da Silva, Maria do Céu Monteiro, Ivani T. de Oliveira, Bruno G. Ferreira

**Affiliations:** 1 Instituto de Biologia, Departamento de Botânica, Universidade Federal do Rio de Janeiro (UFRJ), Rio de Janeiro, RJ CEP 21941-902 Brazil; 2 Departamento de Agronomia , Universidade Federal dos Vales do Jequitinhonha e Mucuri (UFVJM), Diamantina, Brazil; 3 Departamento de Ciências Exatas e Biológicas, Universidade Federal de São João-Del Rei (UFSJ), Campus de Sete Lagoas, Sete Lagoas, MG Brazil

**Keywords:** Blackberry, Rosaceae, Rust on leaves, Anatomy

## Abstract

Pathogenic fungi that cause rust adversely affect plants, including crops, affecting them differentially depending on the cultivar. This is the case of blackberry (*Rubus* spp.), a member of Rosaceae, introduced in Brazil by Embrapa during the 1980s and 1990s. Given the advantageous nutritional properties of its fruits and the relatively low management costs associated with its cultivation, blackberry production has emerged as a significant alternative agricultural source for family farmers. We studied nonsymptomatic leaves of *Rubus* spp. and those infected with *Kuehneola uredinis* to evaluate structural and histochemical alterations induced by the rust fungi and compared the responses among the three blackberry cultivars. *Kuehneola urenidis* colonizes the mesophyll region through hyphal growth, leading to the formation of a pseudoparenchyma and to cell hypertrophy. As colonization progresses, the epidermis becomes ruptured, leading to the production of urediniospores and teliospores. While the cultivars exhibit similar anatomical characteristics, their profiles diverge in the presence of *K. uredinis*. The Guarani cultivar exhibits more intense tissue expansion responses in the presence of the fungus, with an increase in total leaf blade thickness (15%) and adaxial epidermis thickness (25.9%). On the other hand, the BRS Xavante cultivar suffers the greatest structural losses, showing a reduction in mesophyll (9%) and a drastic 40% drop in palisade parenchyma thickness. Finally, the BRS Tupy cultivar proved to be more stable, maintaining better tissue preservation. In regions colonized by *K. uredinis*, the histochemical profile undergoes significant changes, characterized by the accumulation of phenolic compounds, proteins, lipids, and essential oils, compared with those in nonsymptomatic regions, although essential oils were less detected in the more susceptible BRS Xavante. Ultimately, our results confirm varying degrees of susceptibility among the cultivars, providing insights for future research and breeding programs aimed at developing rust-resistant blackberry varieties.

**Supplementary Information:**

The online version contains supplementary material available at 10.1007/s00709-026-02230-w.

## Introduction

Rosaceae encompasses a variety of crops of agronomic importance within the red fruit group, including blackberry (*Rubus* spp.), raspberry (*Rubus occidentalis* and *Rubus idaeus*), blueberry (*Vaccinium corymbosum*), and strawberry (*Fragaria × ananassa*) (Chebotar et al. 2022). Although blackberry cultivation in Brazil is relatively recent compared with that of other crops in this family, its consumption has increased markedly, driven by the recognized nutritional and health benefits of its fruits (Raseira et al. 2020). Blackberry fruits are rich in bioactive compounds, including stilbenes, phenolics, flavonoids, and tannins (Barros et al. 2023; Souza et al. 2014). Moreover, the relatively low cost of orchard establishment, coupled with the high profitability of the crop and the rapid return on investment, position blackberry cultivation as an attractive option in the global market (Gruner and Kornilov 2020).

In Brazil, native *Rubus* species, including *R. urticifolius*, *R. erythrocladus*, *R. brasiliensis*, *R. sellowii*, and *R. imperialis*, occur; however, these species have not been domesticated (Reitz and Klein 1996). Over the years, several commercial cultivars derived from United States (U.S.) breeding programs have been introduced to Brazil (Antunes et al. 2014). Notably, during the 1980s and 1990s, Brazilian cultivars such as Ébano, Negrita, BRS Tupy, Guarani, Caingangue, and BRS Xavante were developed through the Embrapa Clima Temperado breeding program (Antunes 2002; EMBRAPA 2023). The BRS Tupy cultivar is recognized for its resistance to gray mold caused by *Botrytis cinerea* Pers. (Heliotiales). Except for the Ébano and BRS Xavante cultivars, all other cultivars have thorny stems (Raseira et al. 2020). Research has focused primarily on understanding the phenological aspects of these cultivars, as each cultivar shows unique characteristics in its development cycle and chilling requirements for optimal production (Antunes 2002; Oliveira et al. 2017).

Although blackberries are considered hardy due to their adaptability to various climatic and environmental conditions, they are susceptible to a range of viral, bacterial, and fungal diseases that can significantly impact fruit production (Martin et al. 2013; Martinez et al. 2023). Among these, rust fungi (Pucciniales) represent the most diverse group of plant pathogenic fungi and are economically significant diseases affecting blackberry cultivation. These etiological agents can be lethal to the host plant (Aime et al. 2018; Gautheir and Sayre 2021; Oliveira et al. 2021). These agents are obligate biotrophic pathogens that establish a specific relationship with their host plant (Mendgen and Hahn 2002; Migliorini Mendes et al. 2023; Oliveira et al. 2021).

Rust poses a significant challenge to blackberry cultivation across regions worldwide, as this species hosts multiple pathogens responsible for the disease. Notable pathogens include *Kuehneola uredinis* (Link) Arthur (Cheon et al. 2013; Yun 2016; Shands et al. 2018), which causes rust on canes and leaves; *Gymnoconia nitens* (Schwein.) F. Kern and Thurst (Arthur 1917; Morris et al. 1999), responsible for orange rust; and *Phragmidium violaceum* (Schultz) G. Winter (Osterbauer et al. 2005), the agent of blackberry rust (Valeriano et al. 2021). In addition to typical rust symptoms, some fungal pathogens, such as *Kuehneola uredinis*, can induce galls. These fungus-induced galls are more common in eudicotyledons, particularly on leaves, where the tissues of these neoplastic structures often exhibit pronounced alterations compared with the host leaf. The changes that characterize galls include cell hypertrophy and hyperplasia, as well as the disappearance of intercellular spaces, among other modifications (Mani 1964; Migliorini Mendes et al. 2023). Furthermore, these plant‒pathogen interactions can lead to alterations in the host plant histochemical profile, leading to structural and metabolic changes that may benefit herbivores. These responses vary depending on the tissues involved and the primary and secondary compounds accumulated (Migliorini Mendes et al. 2023). The accumulation of primary metabolites is characteristic of each gall-inducing taxon, highlighting their specific nutritional demands (Bragança et al. 2017; Ferreira et al. 2017). For example, in the well-studied analogous systems of insect galls, secondary metabolite accumulation is associated with metabolic events that can protect galling insects (Bragança et al. 2017; Kuster et al. 2020) or prevent oxidative bursts in galled tissues (Kuster et al. 2020).

Plants also actively respond to the presence of pathogens; for example, phenolics are linked to the defensive responses of host plants against some rust fungi (Heath 1997). However, a study on the dynamics of cell wall components and the histochemical profile of a rust fungus gall on *Byrsonima variabilis* A. Juss. demonstrated that the accumulation of phenolic compounds in the tissues did not inhibit spore germination or fungal colonization of the host plant tissues (Migliorini Mendes et al. 2023). Given these findings, we propose that similar dynamics could be observed in infections caused by fungi such as *K. uredinis* in *Rubus* spp. This could lead to anatomical peculiarities and changes in the histochemical profiles of different cultivars affected by cane rust and leaf rust. To investigate this, we evaluated the following hypotheses: (i) *K. uredinis* infection causes specific structural changes in cultivar leaves, promoting the reorganization of the leaf parenchyma to accommodate fungal pustule development; (ii) the three blackberry cultivars exhibit different anatomical responses to fungal infection, resulting in tissue variations and the formation of a protective barrier; and (iii) infection induces the production of specific histochemical compounds, which vary according to the susceptibility levels of the cultivars. This study ultimately aims to understand the interaction between *Rubus* spp. and *K. uredinis* by analysing how the pathogenic agent alters host tissues.

## Materials and methods

### Collection of plant material

**Fig. 1:**
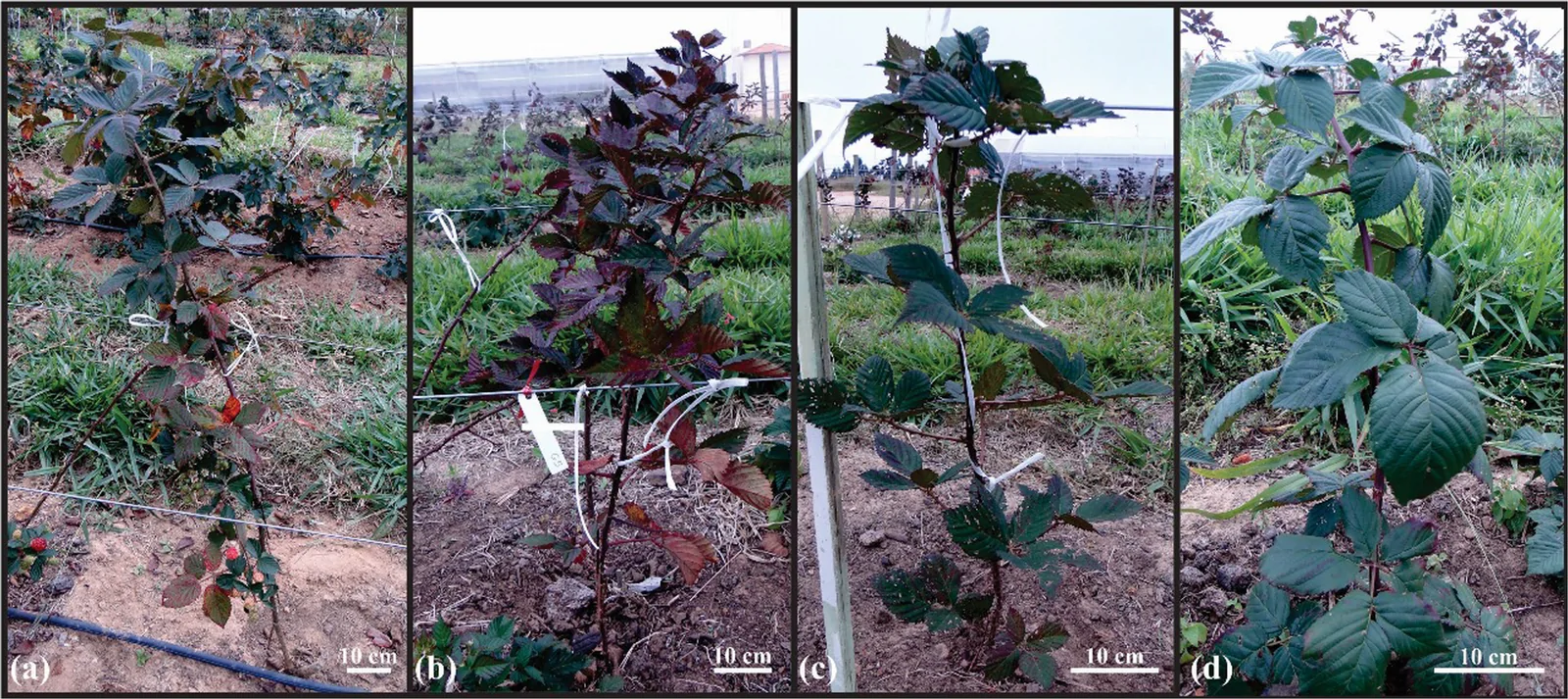
Individuals of *Rubus* spp. cv. Brazos (**a**), Guarani (**b**), BRS Tupy (**c**) and BRS Xavante (**d**) cultivars planted in the Fruit Growing sector of UFVJM

The blackberry (*Rubus* spp.) cultivations studied here were located in the experimental fields at the Fruit Growing Laboratory, Department of Agronomy, Universidade Federal dos Vales do Jequitinhonha e Mucuri (UFVJM), Diamantina, Minas Gerais, Brazil. The site is located at an altitude of 1,387 m at 18°14’56"S, 43°36’00"W. The blackberry (*Rubus* spp.) cultivars Brazos (Fig. 1a), Guarani (Fig. 1b), BRS Tupy (Fig. 1c), and BRS Xavante (Fig. 1d) were planted between 2010 and 2012. These cultivars were supplied by the Empresa Brasileira de Pesquisa Agropecuária (EMBRAPA 2025). Some cultivars are officially registered with the Ministério da Agricultura, Pecuária e Abastecimento (MAPA 2025); specifically, BRS Tupy is registered under number 20,339, and BRS Xavante is registered under 20,384. For this study, we selected Guarani, BRS Tupy, and BRS Xavante because the Brazos variety presented poor-quality leaves and significant defoliation at the time of collection (Martins et al. 2013; Valeriano et al. 2021). The three cultivars analysed are recognized for their susceptibility to rust. Symptomatic regions (affected by *K. uredinis*) and nonsymptomatic regions (free of *K. uredinis*) of infected leaves were collected from the experimental field between December 2023 and March 2024, a period conducive to the emergence of rust in the crop. Dried samples of *Rubus* spp. with *K. uredinis* were deposited in the Herbarium of the Museu Paraense Emílio Goeldi (MG), Belém, Pará State, Brazil, and registered as MG227487 (Valeriano et al. 2021).

### Morphological and anatomical analysis

For morphological characterization, photographic records and phenotypic analysis were performed on the leaves of different blackberry cultivars. For the anatomical study of the Guarani, BRS Tupy, and BRS Xavante cultivars, a portion of the collected samples was fixed in FAA_70_ (37% formaldehyde: glacial acetic acid: 70% ethanol; 1:1:18) (Kraus and Arduin 1997), dehydrated in an increasing ethanol series, embedded in Leica^®^ historesin, and transversely sectioned (5–8 μm) with a rotary microtome. The sections were affixed on glass slides with Bissing’s adhesive (Kraus and Arduin 1997) and dried on a hotplate at 45 °C. The slides were subsequently immersed in 5% cotton blue–lactophenol solution for 5 min on a hot plate. The samples were then washed three times with distilled water (1 min each) to remove excess dye. Finally, they were stained for 10 s in a 1% safranin solution diluted in distilled water and washed again (Marques et al. 2013). The slides were dried and mounted with Entellan^®^ resin, covered with coverslips, and analysed under an Olympus BX51 light microscope. Images were captured with an Olympus SC30 camera.

### Histometric analysis

For histometric analyses, measurements were conducted on symptomatic regions and nonsymptomatic regions of the Guarani, BRS Tupy, and BRS Xavante cultivars with the ImageJ software (Schneider et al. 2012). Transverse sections of leaves from *n* = 3 individuals were used, with three sections per individual and a total of nine replicates per treatment. The evaluated parameters included leaf blade thickness, the thickness of the adaxial and abaxial epidermis, mesophyll thickness, the thickness of the palisade and spongy parenchyma, the number of layers of palisade and spongy parenchyma, and the number of druses per area.

### Histochemical analysis

Fixed and embedded material from affected and unaffected leaf regions of Guarani, BRS Tupy, and BRS Xavante cultivars (*n* = 3) was sectioned at a thickness of 5–8 μm for histochemical analysis. Blank sections were used as negative controls. Histochemical analyses included the following tests: starch detection using Lugol’s solution (Johansen 1940); detection of lipophilic substances with Sudan IV (Jensen 1962); identification of phenolic compounds with 10% ferric chloride (Johansen 1940); protein detection with xylidine ponceau (Vidal 1970); and the use of NADI (α-naphthol and N, N-dimethyl-p-phenylenediamine hydrochloride) for identifying essential oils (David and Carde 1964).

### Statistical analysis

The experimental design employed a randomized factorial arrangement, with factor A comprising three blackberry cultivars (Guarani, BRS Tupy, and BRS Xavante) and factor B consisting of two conditions: symptomatic regions and nonsymptomatic regions of infected leaves. The quantitative data were subjected to normality tests with the Shapiro‒Wilk test (Shapiro and Wilk 1965) and homogeneity of variance tests with Levene’s test (Levene 1960). When necessary, data transformation was performed with the BOXCOX package with R^®^ software (R Core Team 2019). Analysis of variance (ANOVA) was subsequently conducted, and significant differences between means (*p* ≤ 0.05) were further analysed with Tukey’s test at the 5% probability level. The Mantel test was employed to estimate the cophenetic correlation among treatments to assess the distances between treatments and the distances represented in the dendrogram. For this purpose, the frequency and Ward’s methods were utilized to calculate distances between treatments on the basis of nine histometric variables. Additionally, multivariate analysis, including principal component analysis (PCA), was performed to differentiate conditions and evaluate the behavior of individual traits under different conditions via R^®^ software (R Core Team 2019).

## Results

### Morphological characterization

**Fig. 2:**
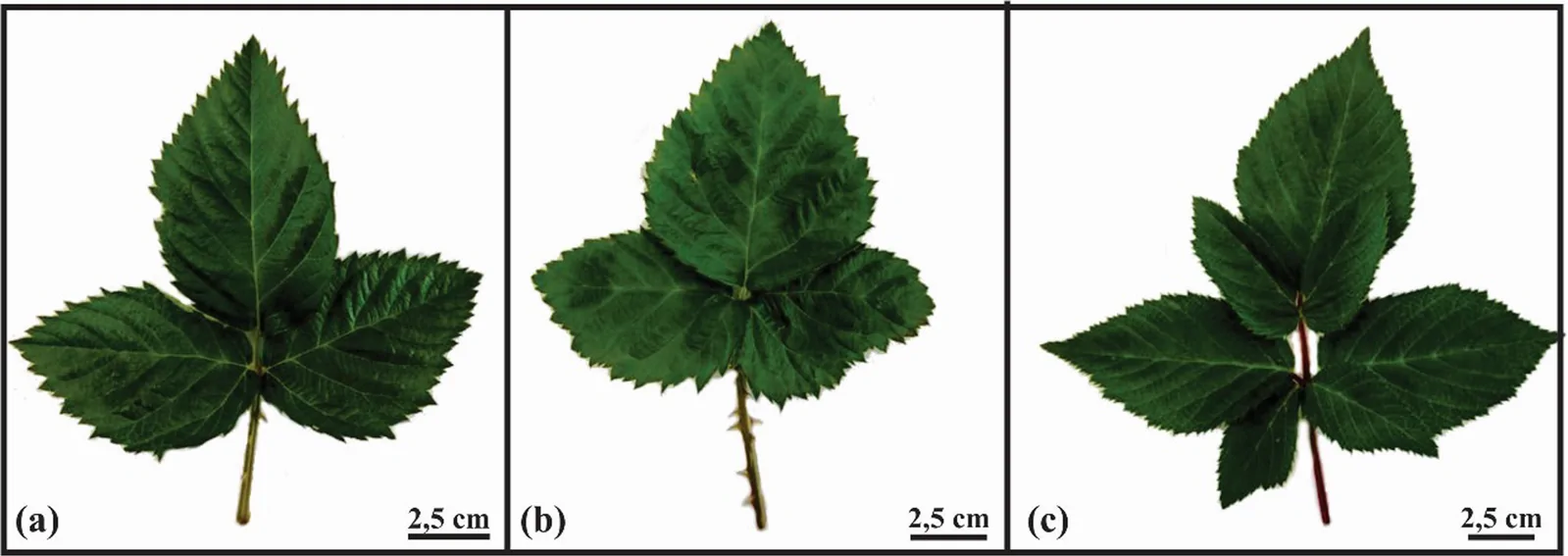
Leaves of *Rubus* spp. cv. Guarani (**a**), BRS Tupy (**b**) and BRS Xavante (**c**)

The leaves of various blackberry cultivars (*Rubus* spp.) presented distinct morphological characteristics. The Guarani (Fig. 2a) and BRS Tupy cultivars (Fig. 2b) presented trifoliate leaves and stems with thorns, whereas the BRS Xavante cultivar presented imparipinnate leaves with seven leaflets and stems without thorns (Fig. 2c). The leaflets of all the cultivars had toothed margins (Fig. 2a-c).

### Anatomical characterization of *Rubu*s spp

**Fig. 3:**
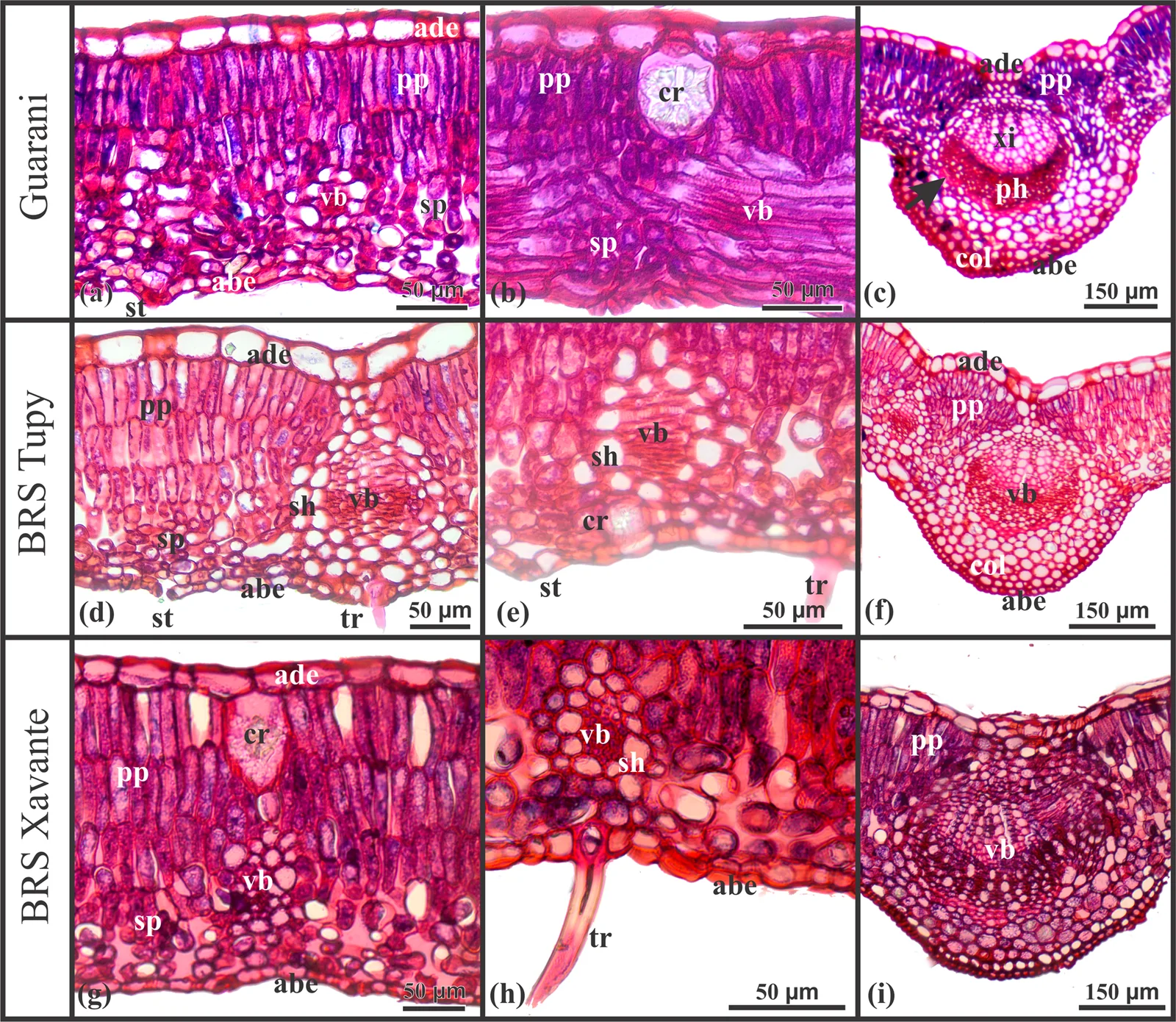
Anatomical transverse sections of leaves of *Rubus* spp. cv. Guarani (**a**–**c**), BRS Tupy (**d**–**f**) and BRS Xavante (**g**–**h**). Abbreviations: ade: adaxial epidermis; abe: abaxial epidermis; col: collenchyma; cr: crystal; ph: phloem; pp: palisade parenchyma; sp: spongy parenchyma; st: stomata; sh: sheath; tr: trichome; vb: vascular bundle; xi: xylem

Anatomically, the three studied cultivars are similar (Fig. 3a-i). The leaf blade epidermis, on both the adaxial and abaxial surfaces, is uniseriate (Fig. 3a, d, g). The adaxial side features rectangular-shaped cells, which are apparently larger than those on the abaxial side (Fig. 3a, d, g). A thin cuticle is present on both epidermal surfaces, along with unicellular, nonglandular, unbranched, conical-shaped trichomes (Fig. 3e, h). The leaves of the three cultivars are hypostomatic, with elevated stomata arranged exclusively on the abaxial side of the leaf (Fig. 3a-b, e, g). The mesophyll is dorsiventral (Fig. 3a-i), with palisade parenchyma located adjacent to the adaxial epidermis (Fig. 3a, d, g). This parenchyma comprises an average of three layers of elongated cells across all cultivars (Table 1). The spongy parenchyma is situated adjacent to the abaxial epidermis, with cells of isodiametric shapes arranged in an average of three layers (Table 1) and few intercellular spaces (Fig. 3a, d, g). In the intermediate zone between the palisade parenchyma and the spongy parenchyma, secondary collateral vascular bundles are present (Fig. 3a, d-e, g-h), surrounded by a sheath of parenchyma cells that extends to both epidermal layers of the leaf blade in the greater secondary bundles. Additionally, idioblasts containing druses are observed in the palisade parenchyma (Fig. 3b, g). Histologically, the midrib consists of an angular collenchyma arranged on both sides (Fig. 3c, f, i), followed by a sheath of parenchymatic cells that surround a single collateral vascular bundle (Fig. 3c, f, i).

**Table 1:** Leaf histometry of *Rubus* sp. cultivars in symptomatic and nonsymptomatic regions after infection by *Kuehneola uredinis*

	Adaxial Epidermis (µm)			Abaxial Epidermis (µm)		
	Guarani	BRS Tupy	BRS Xavante	Guarani	BRS Tupy	BRS Xavante
Nonsymptomatic	0.369 Ab	0.437 Aa	0.423 Aa	0.172 Ab	0.197Ab	0.175 Ab
With *K. uredinis*	0.498 Aa	0.485 Aa	0.403 Ba	0.222 Aa	0.237Aa	0.233 Aa
CV (%)	14.5			25.83		
	Mesophyll (µm)			Palisade Parenchyma (µm)		
Nonsymptomatic	2.891 Bb	3.327 Aa	3.702 Aa	1.956 Ba	2.274 Ba	2.821 Aa
With *K. uredinis*	3.344 Aa	3.387 Aa	3.356 Ab	2.185 Aa	2.142Aa	2.273 Ab
CV (%)	10.05			12.67		
	Spongy Parenchyma (µm)			Number of palisade parenchyma cell layers		
Nonsymptomatic	0.853 Ab	0.897 Ab	0.961 Ab	3.22 Aa	3.22Aa	3.55Aa
With *K. uredinis*	1.180 Aa	1.188 Aa	1.031 Aa	3.44Aa	3.22Aa	3.44Aa
CV (%)	17.36			14.5		
	Number of spongy parenchyma cell layers			Leaf blade (µm)		
Nonsymptomatic	3.77 Ab	3.77 Ab	3.88 Ab	3.387 Cb	3.907 Ba	4.390 Aa
With *K. uredinis*	5.0 Aa	5.0 Aa	4.66 Aa	4.045 Aa	4.141 Aa	4.030 Ab
CV (%)		12.11			8.26	
	Number of druses per area					
Nonsymptomatic	0.33 Aa	0.77 Aa	0.88 Aa			
With *K. uredinis*	0.22 Aa	0.66 Aa	0.0 Aa			
CV (%)		124.16				

Uppercase letters indicate that comparisons should be made in the rows, while lowercase letters indicate that comparisons should be made in columns

*CV* Coefficient of variation

### Anatomy of the interaction between *Rubus* spp. and *Kuehneola uredinis*

**Fig. 4:**
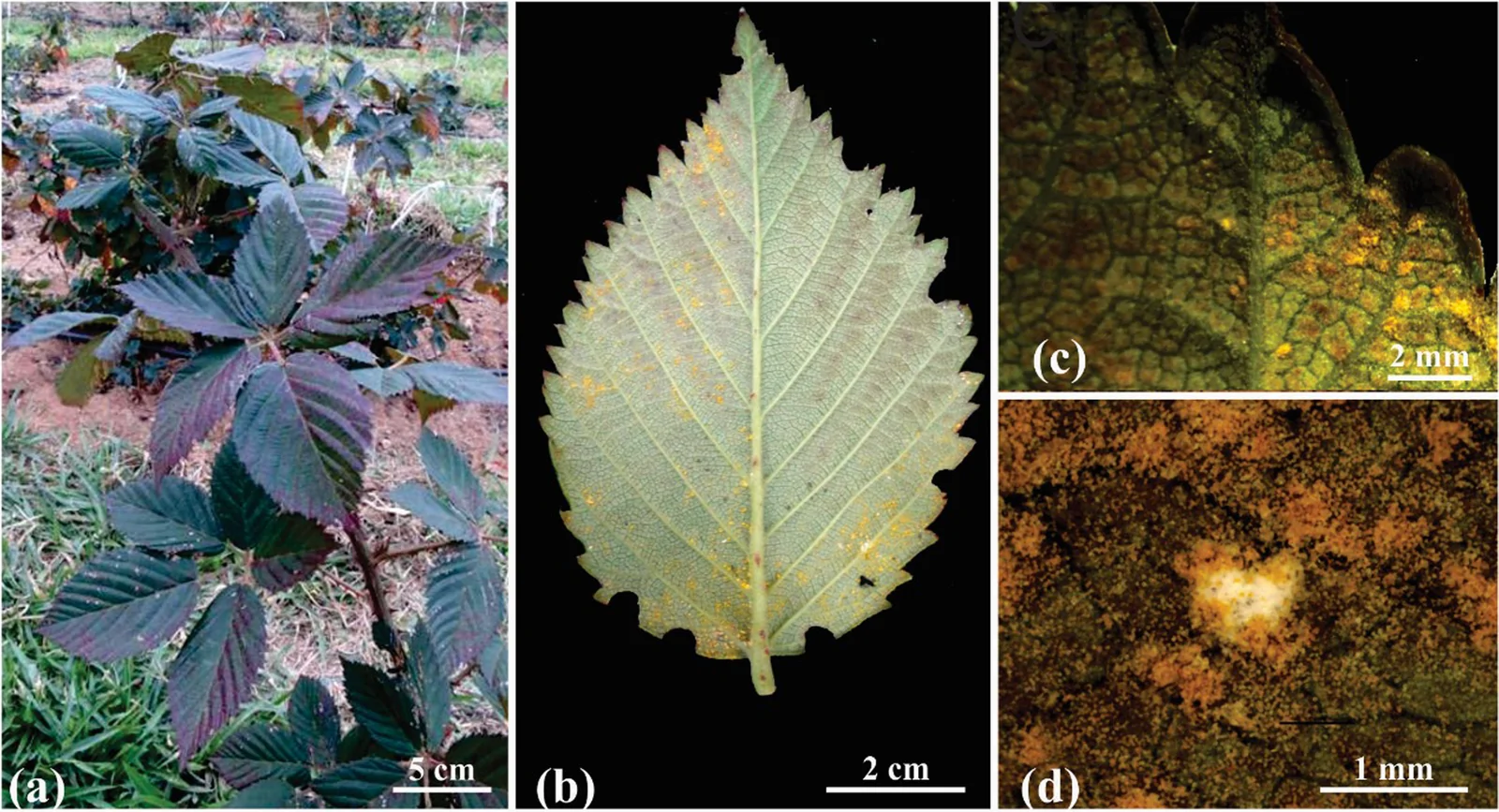
*Rubus* spp. cv. Guarani (**a**) infected with *Kuehneola uredinis* with visual symptoms. Mature leaves showing visual symptoms caused by rust, such as yellow urediniospores and telia containing whitish teliospores on the abaxial surface of the leaf (**b**–**d**)

Fungal sporulation is confined to the abaxial epidermis of the leaves of *Rubus* spp. (Fig. 4a-d), resulting in the formation of pustules containing yellowish globoid urediniospores (Fig. 4b-c). Additionally, whitish to cream subepidermal telia, which contain urediniospores (Fig. 4d), as well as hyaline, cylindrical teliospores with horizontal septa, were common across all the cultivars.

**Fig. 5:**
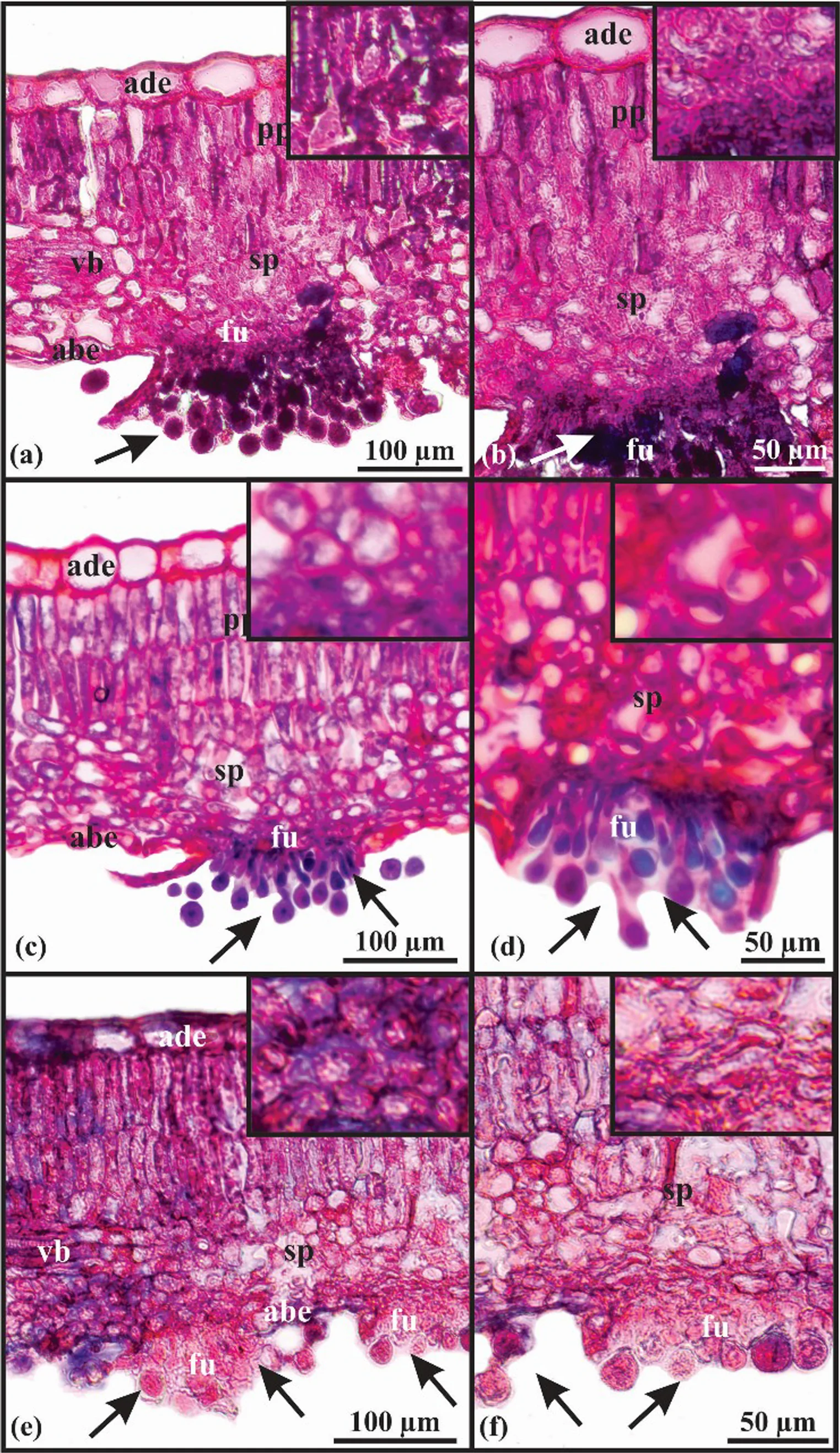
Cross sections of symptomatic regions of *Rubus* spp. cv. Guarani (**a**–**b**), BRS Tupy (**c**–**d**) and BRS Xavante (**e**–**f**) leaves colonized by *Kuehneola uredinis* showing the formation of urediniospores (arrows). Details indicate fungal hyphae stained by cotton blue. Abbreviations: ade: adaxial epidermis; abe: abaxial epidermis; pp: palisade parenchyma; sp: spongy parenchyma; fu: fungi

Common characteristics were noted among the cultivars due to colonization and pustule formation by *K. uredinis* (Fig. 5a-f). Rust develops intralaminarly from the germination of urediniospores, primarily on the adaxial surface of the *Rubus* spp. leaf, entering through the epidermis towards the mesophyll, where the hyphae proliferate and occupy the palisade and spongy parenchyma (Fig. 5a, c, e). The hyphae and host plant cells form a pseudoparenchyma (Fig. 5b, d, e). The mesophyll appeared more compact, with fewer spaces between the cells, as the hyphae occupied the intercellular spaces (Fig. 5b, d, e). On the abaxial surface, the epidermal layer is separated and fully opened following rupture and detachment from the spongy parenchyma caused by the paraphyses and spores (Fig. 5a, d, f). Additionally, *K. uredinis* did not colonize the vascular bundles, the midrib or the secondary veins.

### Histometry of symptomatic regions and nonsymptomatic regions

The histometric features of the *Rubus* spp. leaf tissues of the cultivars Guarani, BRS Tupy, and BRS Xavante were significantly different among the symptomatic and nonsymptomatic regions of leaves infected by *K. uredinis* (Table 1). Notable differences were observed in the thickness of the epidermis and mesophyll as well as in the spongy and palisade parenchyma, allowing us to infer variations in the susceptibility of each cultivar to the pathogen. Compared with nonsymptomatic regions, the Guarani cultivar presented a 25.9% increase in the thickness of the adaxial epidermis in the symptomatic region (Table 1). In contrast, the BRS Tupy and BRS Xavante cultivars did not significantly differ in the thickness of the adaxial epidermis between treatments. However, the symptomatic area of the BRS Xavante cultivar had a thinner adaxial epidermis (ca. 20%) than did the symptomatic area of the other cultivars. With respect to the abaxial epidermis, no significant differences were observed among the nonsymptomatic regions in all the cultivars; however, an increase of ca. 20% in thickness was recorded in response to the presence of the fungus in all cultivars when compared to the nonsymptomatic regions.

While the presence of *K. uredinis* led to an increase in mesophyll thickness in the Guarani cultivar (ca. 15%) and a reduction in the BRS Xavante cultivar (ca. 9%), the BRS Tupy cultivar maintained mesophyll thickness with fungal infection (Table 1). The thickness of the palisade parenchyma remained similar in Guarani and BRS Tupy cultivars after *K. uredinis* infection, whereas the BRS Xavante cultivar presented a 40% reduction in palisade parenchyma (Table 1). In the nonsymptomatic regions, the palisade parenchyma thickness in the BRS Xavante cultivar was ca. 40% greater than that in the Guarani and BRS Tupy cultivars (Table 1). With respect to the number of palisade parenchyma cell layers, no significant differences were observed among the cultivars or between the presence and absence of the pathogen (Table 1). The presence of *K. uredinis* resulted in an approximately 25% increase in the thickness of the spongy parenchyma across all the cultivars (Table 1). In addition, the number of spongy parenchyma cell layers increased by ca. 20% in all cultivars due to fungal infection (Table 1). At the sites of *K. uredinis* infection, the leaf blade thickness increased by ca. 15% in the Guarani cultivar, while the BRS Tupy cultivar maintained the leaf blade thickness, and the BRS Xavante cultivar had a reduction of ca. 8% (Table 1). In the absence of the pathogen, the average leaf blade thickness was greater in the BRS Xavante cultivar, followed by BRS Tupy, and the thinnest leaf blade was observed in the Guarani cultivar (Table 1). The number of druses observed was 40% greater in Guarani and 20% greater in BRS Tupy than in BRS Xavante, although the treatments were not significantly different (Table 1).

To better visualize the results of the univariate analysis (Table 1), the multivariate analyses (Online Resources 1–3) confirm that *K. uredinis* infection induces distinct structural modifications, clearly separating the symptomatic from the nonsymptomatic regions. The Guarani cultivar presented the greatest dissimilarity between nonsymptomatic and symptomatic regions, reflecting the increment observed in several structures (Online Resources 1 and 2). Principal component analysis (PCA) revealed that this clear separation in Guarani is primarily explained by variations in the thickness of the mesophyll, leaf blade, and palisade parenchyma (Online Resource 3a). In contrast, the BRS Tupy cultivar demonstrated a less pronounced reaction, with greater overlap between the groups (Online Resource 3b). As for the BRS Xavante cultivar, which was the most negatively affected by the disease, the separation was associated with different variables, notably the abaxial epidermis and spongy parenchyma (Online Resource 3c).

### Histochemistry of *Rubus* spp. leaves

**Fig. 6:**
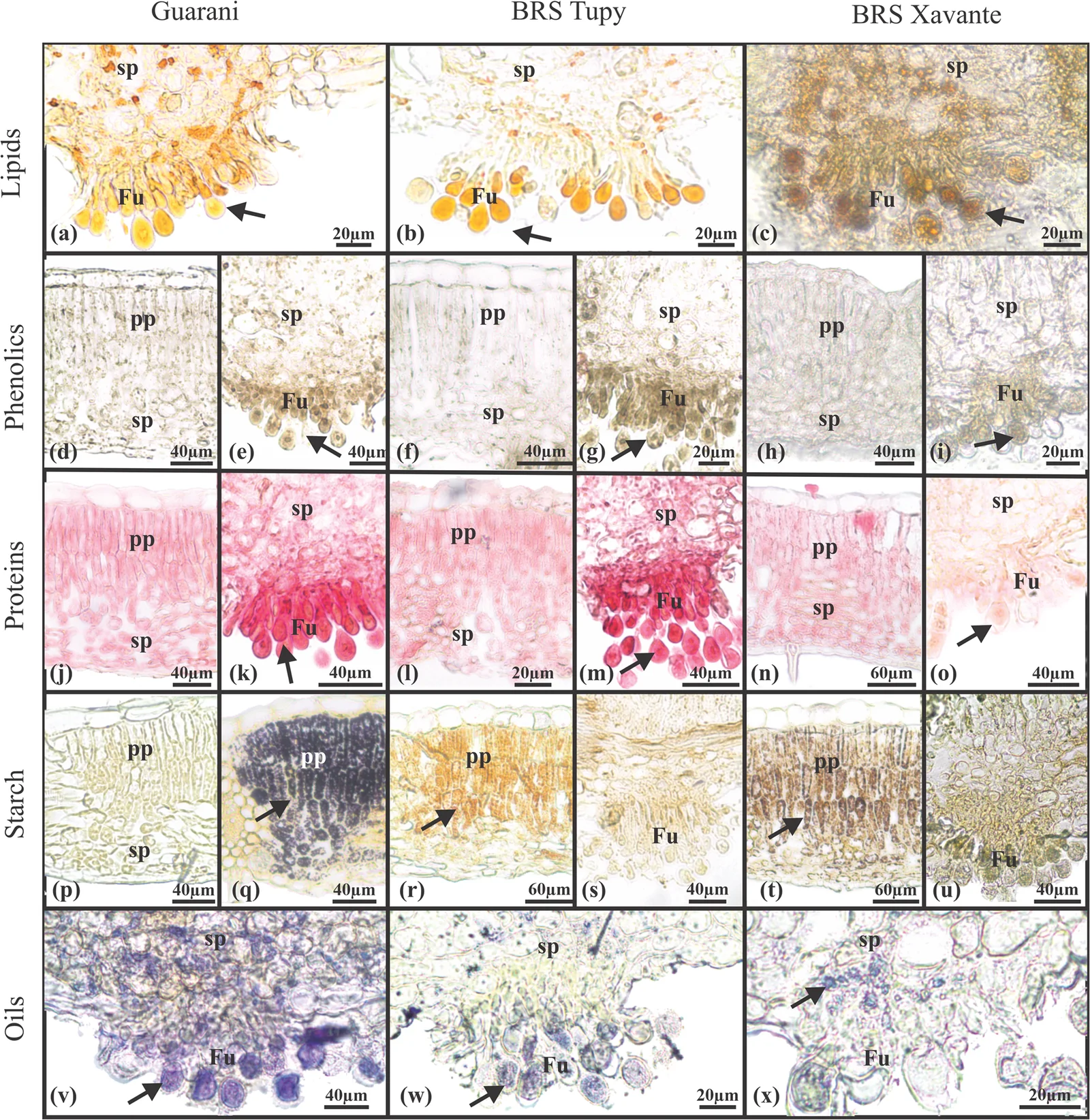
Histochemical analysis of the nonsymptomatic and symptomatic regions of the leaves of *Rubus* spp. cv. Guarani (**a**, **d**, **e**, **j**, **k**, **p**, **q**, **v**), BRS Tupy (**b**, **f**, **g**, **l**, **m**, **r**, **s**, **w**) and BRS Xavante (**c**, **h**, **i**, **n**, **o**, **t**, **u**, **x**). Lipids detected in the symptomatic regions of the three cultivars (**a**–**c**). Phenolic compounds with high intensity in the urediniospore region (**e**, **g**, **h**). Proteins with low intensity in the leaf mesophyll (**j**, **l**, **n**) and high intensity in the urediniospore region (**k**, **m**). Starch was absent in some regions of the leaf mesophyll (**p**), including colonization areas (**s**, **u**), and was present in some regions of the leaf mesophyll (**q**, **r**, **t**). Concentration of essential oils with greater intensity in the symptomatic regions of Guarani and BRS Tupy cultivars (**v**–**w**) and moderately in the BRS Xavante cultivar (**x**)

Lipids were detected in the cuticle of the adaxial and abaxial surfaces of the epidermis in the symptomatic regions of Guarani (Fig. 6a), BRS Tupy (Fig. 6b), and BRS Xavante cultivars (Fig. 6c). Phenolic compounds were not detected in the nonsymptomatic regions of Guarani leaves (Fig. 6d), whereas they were present in the sporulation region of the fungus (Fig. 6e). A similar pattern was observed in the BRS Tupy (Fig. 6f-g) and BRS Xavante (Fig. 6h-i) cultivars. Proteins were detected at a lower intensity in the palisade and spongy parenchyma of nonsymptomatic Guarani leaves (Fig. 6j). In contrast, a visually higher intensity of protein was observed in the sporulation region of the fungus (Fig. 6k). The same occurred for the BRS Tupy cultivars (Fig. 6l-m). In contrast, the BRS Xavante cultivar presented a lower intensity of proteins in the nonsymptomatic leaves and in the sporulation region of the fungus (Fig. 6n-o). Small starch grains were identified in the palisade parenchyma cells and, more discretely, in the spongy parenchyma (Fig. 6q, r, t). In the sporulation region of the fungus, starch grains were not detected (Fig. 6s, u). Essential oil droplets were observed at a visually lower intensity in the palisade and spongy parenchyma of the nonsymptomatic regions of the Guarani, BRS Tupy, and BRS Xavante cultivars. In contrast, they were detected at a visually strong intensity in the region of fungal growth in the Guarani and BRS Tupy cultivars (Fig. 6v-w) and a moderate intensity in the BRS Xavante cultivar (Fig. 6x).

## Discussion

Our study revealed the structural and histochemical mechanisms of three distinct blackberry cultivars in response to interactions with *K. uredinis*, the fungus responsible for cane and leaf rust in blackberry. Our structural, histochemical, and histometric study of the effects of *K. uredinis* infection on *Rubus* spp. demonstrated that the infection causes specific structural changes in cultivar leaves and promotes reorganization of the leaf parenchyma to accommodate fungal pustule development. The three blackberry cultivars presented slightly distinct histometrical responses to fungal infection, which were most pronounced in the BRS Xavante cultivar. Finally, we demonstrated that fungal infection induces the production of specific histochemical compounds, such as proteins, phenolics, and essential oils. This accumulation was more pronounced in the Guarani and BRS Tupy cultivars.

The general leaf morphology of the cultivars studied here is similar to that previously reported for species and varieties within the Rosaceae family, such as mature leaves of *Rubus idaeus* L. cv. Autumn Bliss, *Rubus occidentalis* L. and *Rubus niveus* Thunb. (Dias et al. 2023). Additionally, the number of leaflets, ranging from three to seven, aligns with what has been documented for the genus *Rubus* (Dias et al. 2023; Fell and Rowson 1956, 1961). When the anatomical aspects of the leaves of blackberry cultivars were compared, the varieties presented similar structural characteristics, including the organization and arrangement of the tissues and the presence of calcium oxalate. However, differences were noted in certain aspects, such as the size of the mesophyll and palisade parenchyma, which can be utilized to distinguish between cultivars.

The presence of nonglandular trichomes was observed on the leaf epidermis of different cultivars, with variations in size. This characteristic was also observed in three raspberry species (Dias et al. 2023). Although these epidermal structures can serve as physical defenses against herbivores (Karley et al. 2016), research has shown that they do not act as a fully effective physical barrier against fungal infections and that a high density of trichomes is a factor that favours infection (Dias et al. 2023; Wang et al. 2020).

Blackberry lacks stomata in the adaxial epidermis, and its varieties are hypostomatic. Previous studies have also reported hypostomatic leaves of *Rubus plicatus* Weihe & Nees, *R. idaeus*, and *R. loganobaccus* (Dias et al. 2023; Petrus-Vancea et al. 2024). This characteristic has been recognized as a significant adaptation, enabling these species to thrive in various environments (Petrus-Vancea et al. 2024), although this may represent the critical pathway for the infection of foliar pathogens into the internal tissues of the host (Muir 2020; Dias et al. 2023; Solanki et al. 2019).

Here, we propose that the behavior of *K. uredinis* in blackberry leaves is similar to that of the basic model reported for some fungi infecting plant tissues (Arya and Cohen 2022; Cooper et al. 2016). Spores of *K. uredinis* land predominantly on the adaxial surface of blackberry leaves, attaching themselves to the surface using adhesive compounds. Because there is greater moisture accumulation on the adaxial surface, the spores hydrate by absorbing water and then germinate. Fungal penetration into the plant must occur through specialized hydrolytic enzymatic activities, such as cutinases, which degrade cutin, allowing access through the intact epidermis to colonize the host plant’s internal cells, or, more easily, through natural cracks or wounds that facilitate entry (Arya and Cohen 2022; Cooper et al. 2016). Following penetration, *K. uredinis* hyphae develop and predominantly occupy the intercellular spaces of the spongy parenchyma, particularly near the substomatal chamber, where they form a pseudoparenchyma. The formation of such pseudoparenchyma has also been reported in plants infected by rust fungi (Pucciniales) (Heller 2020).

Tissue changes such as hyperplasia (increased cell division) and hypertrophy (increased cell size) commonly occur as a result of inducer stimuli. The formation of a pseudoparenchyma appears to allow cell separation to accommodate hyphal growth, increase the number of intercellular spaces, and cause dissolution of the middle lamella of mesophyll cells. The formation of this structure supports our initial hypothesis (i) that *K. uredinis* triggers specific structural changes in *Rubus* spp. leaves, remodelling the parenchymatic tissue to accommodate the development of fungal pustules. This strategy of increasing intercellular spaces and consequently colonizing them reduces communication between plant cells, which appears to hinder the triggering of defense responses in the host plant (Migliorini Mendes et al. 2023).

Our second hypothesis (ii) is also confirmed by the histometric comparison between symptomatic and nonsymptomatic regions, demonstrating that fungal colonization induces not only distinct anatomical responses among cultivars, but that these variations appear to be directly linked to the ability of each genotype to establish structural defense mechanisms against *K. uredinis*. The increase in the epidermis and mesophyll layer, for example, may be a defensive response related to rapid cell growth and division in the presence of haustoria formed by pathogenic fungi (Kārkliņa et al. 2021). Cell hypertrophy, which is primarily concentrated in the spongy parenchyma, has also been reported for *Vitis vinifera* cv. Moscato Giallo inoculated with *Phakopsora euvitis*, which causes grapevine rust (Navarro et al. 2019). The BRS Xavante cultivar, with a thicker mesophyll, presented a reduction in mesophyll and spongy parenchyma thickness after infection, whereas Guarani presented increased thicknesses, and BRS Tupy maintained this histometric feature.

The reduction in the tissue thickness in the BRS Xavante cultivar appears to be a result of immediate lesions caused by the intercellular growth of the fungus, where the pathogen’s hyphae spread in the mesophyll, invade the space between cells, and cause compression and damage to the structural integrity of the parenchyma, as reported in *Chrysanthemum morifolium* leaves infected with *Puccinia horiana* (Selis et al. 2024). Furthermore, physiological disturbances such as cell wall degradation from the release of digestive enzymes cause cell collapse and lysis, leading to tissue disorganization and atrophy, resulting in reduced thickness and functionality of the photosynthetic tissue (Selis et al. 2024).

Conversely, the palisade parenchyma had apparently more compacted layers, with elongated, side-by-side cells, which were not affected by fungal infection in the cultivars Guarani and BRS Tupy, but was affected in BRS Xavante, which initially (in nonsymptomatic region) had thicker palisade parenchyma compared to the other cultivars. The arrangement of the palisade parenchyma appears to limit the intercellular space available for hyphal growth, which could explain the lower visualization of fungal structures in this tissue when compared to the spongy parenchyma, a phenomenon also reported in a study involving the *Rubus* spp.–*A. americanum* pathosystem (Dias et al. 2023). These quantified histometric variations are significant, as the thickness of the palisade and spongy parenchyma can serve as an important defense mechanism in rust-resistant plants compared with susceptible genotypes (Li et al. 2018). These results indicate that the BRS Xavante cultivar is more affected by *K. uredinis* infection than the other studied cultivars are. Tissue variations were used as morphological markers to distinguish resistant from susceptible genotypes in pear (*Pyrus communis* L.) leaves infected by the European rust *Gymnosporangium sabinae* (Dicks.) Oerst. (Kārkliņa et al. 2021). Therefore, it is necessary to conduct further studies with the parameters analysed to obtain more conclusive answers. Our results also indicate that the main bundle of the midrib and the secondary vascular bundles restrict the advance of *K. uredinis*, acting as a potential barrier against the colonization of the pathogen. Similar behavior was observed in other *Rubus* species and *Vitis vinifera* cultivars (Dias et al. 2023; Navarro et al. 2019). This response may be associated with the limited production of enzymes that degrade lignin, thereby hindering the ability of fungi to colonize this region (Bellincampi et al. 2014).

Multivariate analyses reinforced the histometric differences, indicating anatomical discordances between nonsymptomatic and symptomatic regions of leaves of the distinct cultivars. The dendrogram clustered the nonsymptomatic regions of BRS Tupy and Guarani, setting them apart from the BRS Xavante cultivar even before infection. Principal Component Analysis (PCA) further supported this, showing that specific variables—notably the thickness and number of palisade and spongy parenchyma cells, along with adaxial epidermis, mesophyll, and leaf blade thickness—were key drivers differentiating infected from nonsymptomatic regions, especially defining the responses in Guarani and BRS Xavante. Conversely, the BRS Tupy cultivar displayed greater overlap between symptomatic and nonsymptomatic groups in the PCA, aligning with histometric data suggesting it had the least pronounced morphoanatomical reaction among the three cultivars studied. These combined multivariate findings strongly support the hypothesis that the morphoanatomical response to *K. uredinis* varies significantly among cultivars, correlating with their respective degrees of susceptibility or tolerance.

In addition to the histometric changes, the histochemical results indicated the production of biologically active compounds in the symptomatic regions, both on host plant cells and on fungal cells, confirming our third hypothesis (iii) that the chemical response is modulated by the susceptibility level of each cultivar. This modulation was evidenced by tissue homeostasis and the efficiency of the defensive response to colonization by *K. uredinis*. The distinction was based on the magnitude of structural loss (e.g., 40% reduction in palisade parenchyma in BRS Xavante vs. preservation in BRS Tupy) and the intensity of secondary metabolite accumulation, such as phenolics and essential oils, as markers of defensive efficiency. While more tolerant cultivars mobilized histochemical compounds and showed greater tissue stability, the most susceptible cultivar showed greater tissue degradation. The presence of lipids in histopathological studies has been associated with the activation of various plant defense mechanisms as a protective response against pathogenic organisms. These compounds are transported to repair damaged structures such as cell walls and membranes (Casassola et al. 2015). The cultivar IAC-13 Lorena (*Triticum aestivum*) has been reported to be more sensitive to leaf rust (*Puccinia triticina*), primarily because of the greater detection of lipids and flavonoids in the leaf tissue (Veiga et al. 2020).

Phenolic compounds, which are not detected in nonsymptomatic regions, accumulate in symptomatic regions in response to fungal infection in the three cultivars. High accumulation of these compounds is naturally found in certain wild blackberry species, such as *R. perrobustus*, *R. wimmerianus*, *R. pedemontanus*, *R. grabowskii*, and black raspberries (Oszmiański et al. 2015). However, significant increases in phenolic compounds are reported following infections by phytopathogenic agents such as coffee rust caused by *Hemileia vastatrix* (Carvalho 1991). These elevated levels are associated with efficient defense mechanisms in *Rubus occidentalis* L., which is considered more resistant, and *Rubus niveus* Thunb., which is considered immune to infection by *A. americanum* (Dias et al. 2023). The accumulation of phenolics is a response mechanism against *K. uredinis* infection, although this mechanism is not enough to prevent fungal infection in the blackberry cultivars studied herein, as also observed in coffee cultivars susceptible to coffee rust (Venktasubbaiah et al. 1983). Polyphenols contribute to controlling the increase in reactive oxygen species (ROS), allowing the maintenance of oxidative homeostasis. These phenolic compounds exhibited antioxidant and photoprotective functions in *Ditylenchus gallaeformans* (Nematoda: Anguinidae) galls on *Miconia albicans* (Sw.) Steud., forming an important apparatus against excessive oxidation due to light, and the intense activity of gall-inducing organisms (Dias et al. 2013; Ferreira et al. 2018). In animal-induced galls, phenolic compounds accumulate surrounding galling agents as a mechanism of antioxidant defense, which is important for impairing host cell senescence and maintaining cell survival in the presence of gall inducers (Dias et al. 2013; Isaias et al. 2015; Oliveira et al. 2016), which could be evaluated for *K. uredinis–Rubus* infections.

While starch was reduced in the fungus-colonized regions across all cultivars, the protein content was greater in regions affected by *K. uredinis*. Jubault et al. (2013) reported that infection of *Plasmodiophora brassicae* roots in *Arabidopsis thaliana* promoted changes in the expression of several genes involved in primary metabolism, including those involved in the expression of starch-degrading enzymes, suggesting that this is an advantageous strategy for the pathogen to meet nutrient demands. Thus, the supply of primary metabolic resources, such as carbohydrates, can affect the interaction between pathogens and hosts, promoting distinct plant susceptibility or resistance responses to fungi (Gkizi et al. 2015). One study demonstrated that starch-depleted *A. thaliana* mutants were classified as more resistant to *Erysiphe cruciferarum*, a biotrophic fungus, and showed greater susceptibility to the hemibiotrophic *Colletotrichum higginsianum* (Engelsdorf et al. 2013). Rust fungi secrete a range of effector proteins into host plant tissues to overcome barriers such as the cuticle and promote infection. During colonization, developed structures such as haustoria and infection hyphae transfer these proteins to neutralize or suppress immune responses and obtain nutritional benefits by manipulating the host’s physiological processes (Bradley et al. 2022; Petre et al. 2014).

Essential oils were detected particularly in infected *Rubus* leaves in relation to nonsymptomatic regions, which presented a lower intensity. Essential oil accumulation is a characteristic that may be intrinsic to the host plant (Rambaran and Ginigini 2020). However, interactions with organisms can trigger a series of responses and alter the host’s histochemical profile (Ferreira and Guedes 2025). Colonization by *K. uredinis* altered the histochemical profile among the cultivars, with greater intensity observed in Guarani and BRS Tupy. Although there are few reports on the production of these compounds during plant‒fungal interactions, terpenoids are generally recognized for their antifungal and antibacterial activities and their antioxidant and defensive properties (Delaquis et al. 2002; Dorman and Deans 2000; Francio et al. 2025).

## Conclusions

This study reveals, for the first time, the effects of leaf tissue colonization by *K. uredinis* in different *Rubus* spp. cultivars through structural and histochemical analyses. The results confirmed the susceptibility of all the cultivars to the fungus, which, upon colonizing the tissues, promoted tissue reorganization to accommodate fungal structures. The presence of the fungus causes structural damage among cultivars; however, differences were observed between them, with the BRS Xavante cultivar being more sensitive to the presence of the pathogen than the Guarani and BRS Tupy cultivars are. The BRS Tupy cultivar demonstrated greater tissue stability in the presence of the pathogen through histometric parameters, suggesting more effective structural mechanisms to contain the infection than the other cultivars do. Moreover, fungal-induced changes promote the production of compounds such as proteins, suggesting that these compounds may act as important nutritional sources to support fungal establishment and as neutralizers of the defense mechanisms of *Rubus* spp. Furthermore, the histochemical profile also indicates the accumulation of phenolic compounds and essential oils, which could act in plant defense and as antioxidants to mitigate oxidative stress, especially in the Guarani and BRS Tupy cultivars. Finally, our results highlight the plasticity observed among these cultivars, which provides a basis for future studies aimed at developing rust-resistant blackberry cultivars.

## Supplementary Information

Below is the link to the electronic supplementary material.

Online Resource 1 Frequency of discordance between nonsymptomatic regions and symptomatic regions of *Rubus* spp. cultivars in the presence of rust. T1 = Nonsymptomatic regions of Guarani cultivar; T2: Symptomatic regions of Guarani cultivar; T3: Nonsymptomatic regions of BRS Tupy cultivar; T4: Symptomatic regions of BRS Tupy cultivar; T5: Nonsymptomatic regions of BRS Xavante cultivar; T6: Symptomatic regions of BRS Xavante cultivar (PNG 84.7 KB)

High Resolution Image (TIF 100 KB)

Online Resource 2 Histometric changes in *Rubus* spp. cultivars under rust incidence. Dendrogram formed via Ward’s method. The cut-off criterion is k=1.25. T1 = Nonsymptomatic regions of Guarani cultivar; T2: Symptomatic regions of Guarani cultivar; T3: Nonsymptomatic regions of BRS Tupy cultivar; T4: Symptomatic regions of BRS Tupy cultivar; T5: Nonsymptomatic regions of BRS Xavante cultivar; T6: Symptomatic regions of BRS Xavante cultivar (PNG 27.2 KB)

High Resolution Image (TIF 22.4 KB)

Online Resource 3 Principal component analysis based on histometric changes in tissues of symptomatic regions and nonsymptomatic regions of leaves of *Rubus* spp. cultivars. A: Guarani; B: BRS Tupy; C: BRS Xavante (PNG 672 KB)

High Resolution Image (TIF 338 KB)

Supplementary Material 4 (DOCX 15.5 KB)

## Data Availability

The datasets generated during the current study are available from the corresponding author upon request.
