# Supplementary material for: Histochemical and histometrical changes induced by the rust fungi Kuehneola uredinis in distinct blackberry (Rubus spp.) cultivars
Source: Protoplasma. 2026 Jun 17;263(5):1833–47. doi: 10.1007/s00709-026-02230-w (PMC13493478; doi:10.1007/s00709-026-02230-w)
Supplement: Supplementary file 1 [file 709_2026_2230_MOESM4_ESM.docx]

**Table 2:** ANOVA for the histometric variables in leaf tissue of symptomatic regions and nonsymptomatic portions of *Rubus* spp. cultivars.

|  | **Cultivars * *K. uredinis*** | | | **Cultivars** | | | **Pathogenicity** | | |
| --- | --- | --- | --- | --- | --- | --- | --- | --- | --- |
|  | **SS** | **MS** | ***P*** | **SS** | **MS** | ***P*** | **SS** | **MS** | ***P*** |
| **Adaxial epidermis thickness** | 0.045 | 4 | 0.006^*^ | 0.02 | 3 | 0.06 ^ns^ | 0.039 | 5 | 0.003^*^ |
| **Abaxial epidermis thickness** | 0.00077 | 2 | 0.873^ns^ | 0.0038 | 3 | 0.510 ^ns^ | 0.329 | 5 | 0.001^*^ |
| **Mesophyll thickness** | 1.4361 | 4 | 0.003* | 1.539 | 5 | 0.002^*^ | 0.042 | 2 | 0.542 ^ns^ |
| **Palisade parenchyma thickness** | 1.3594 | 4 | 0.0008^*^ | 2.1621 | 5 | 0.00003^*^ | 0.3053 | 3 | 0.061 ^ns^ |
| **Spongy parenchyma thickness** | 0.1805 | 4 | 0.065^*^ | 0.0183 | 2 | 0.747 ^ns^ | 0.703 | 5 | 0.00002^*^ |
| **Number of palisade**  **parenchyma cells** | 0.2593 | 3 | 0.5811 ^ns^ | 0.7037 | 5 | 0.2355 ^ns^ | 0.0185 | 2 | 0.7806 ^ns^ |
| **Number of spongy**  **parenchyma cells** | 0.593 | 3 | 0.675 ^ns^ | 0.148 | 2 | 0.906 ^ns^ | 15.57 | 5 | 0.0004^*^ |
| **Leaf blade thickness** | 2.3521 | 5 | 0.0001^*^ | 2.2352 | 4 | 0.0018^*^ | 0.423 | 3 | 0.0539 ^ns^ |
| **Number of druses per area** | 1.8148 | 3 | 0.198 ^ns^ | 1.81 | 3 | 0.198 ^ns^ | 1.85 | 4 | 0.070^*^ |

Abbreviations: *SS* Sum of squares, *MS* Mean square, *P* *P-*value, *ns* nonsignificant*, ** significant.
